# Doubly Constrained Waveform Optimization for Integrated Sensing and Communications

**DOI:** 10.3390/s23135988

**Published:** 2023-06-28

**Authors:** Zhitong Ni, Andrew Jian Zhang, Ren-Ping Liu, Kai Yang

**Affiliations:** 1 School of Electrical and Data Engineering, University of Technology, Sydney, NSW 2007, Australia; 2 School of Information and Electronics, Beijing Institute of Technology, Beijing 100081, China

**Keywords:** integrated sensing and communication (ISAC), radar communications, waveform optimization, threshold constraint

## Abstract

This paper investigates threshold-constrained joint waveform optimization for an integrated sensing and communication (ISAC) system. Unlike existing studies, we employ mutual information (MI) and sum rate (SR) as sensing and communication metrics, respectively, and optimize the waveform under constraints to both metrics simultaneously. This provides significant flexibility in meeting system performance. We formulate three different optimization problems that constrain the radar performance only, the communication performance only, and the ISAC performance, respectively. New techniques are developed to solve the original problems, which are NP-hard and cannot be directly solved by conventional semi-definite programming (SDP) techniques. Novel gradient descent methods are developed to solve the first two problems. For the third non-convex optimization problem, we transform it into a convex problem and solve it via convex toolboxes. We also disclose the connections between three optimizations using numerical results. Finally, simulation results are provided and validate the proposed optimization solutions.

## 1. Introduction

Communication and radar are merging into a unified system, known as an integrated sensing and communications (ISAC) system. Radar and communication share many common features in terms of hardware modules and signal processing methods [1,2,3]. Additionally, an integrated system is conducive to improving overall system performance and spectrum sharing [4].

Joint waveform optimization is an important problem in ISAC. For ISAC waveform optimization, many works have been conducted by considering various performance metrics. For radar sensing, a typically considered performance metric is mutual information (MI) [5,6]. In [5], the authors studied MI for a wideband ISAC system, and maximized the weighted sum of the MI of radar and the MI of communications. In [6], the authors developed a combined MI criterion that designs the waveform and power allocation, and optimized the joint performance metric of both radar and communications. For communications, the signal-to-noise interference ratio (SINR) that shows a non-convex feature is commonly used as the optimization goal. The authors in [7] allocated multiple users to orthogonal subcarriers, thereby avoiding multi-user interference (MUI), and the SINR becomes a convex metric. F. Liu et al. proposed to use one weighting factor to sum the independent optimal communication waveform and the independent optimal radar waveform [8]. This scheme is based on the Euclidean distance between the waveforms, and the optimality cannot be guaranteed. Note that all these works adopted simplified expressions of metrics since both MI and SINR have complicated expressions that lead to challenging ISAC waveform optimization problems.

Recently, some solutions have been proposed to optimize the non-convex metrics in the multi-user ISAC systems [9,10,11,12]. These works use multiple thresholds to constrain either radar or communication metrics. In [9], the authors maximized the radar SINR with a given specific capacity of communication channels. The work in [10] further introduced a sub-sampling matrix for radar as an objective function of the optimization. In [11], the authors separated the transmit antenna array into two sub-arrays and optimized the radar performance by adjusting the SINR threshold of communications. Given the thresholds of either radar or communication, this kind of method maximizes one radar/communication performance metric subject to the constraints of the other performance metric.

In this paper, we propose three novel optimization methods that solve the ISAC optimization problems when constraining communication only, constraining radar only, and constraining ISAC performance metrics, respectively. The radar and communication optimization metrics are mutual information and the multi-user sum rate (SR), respectively. The optimality of the proposed optimization method is analyzed via simulations. The contributions of this paper are summarized below:

- We optimize the SR of communications subject to the constraint of radar MI. We exploit the geometric property of MI to determine the moving direction of precoder and use gradient descent optimization methods to optimize the SR.
- We optimize the radar MI subject to the constraint of SR of communications. We exploit the geometric property of SR to determine the moving direction of precoder and use gradient descent optimization methods to optimize the MI.
- We optimize the Euclidean distance between the ISAC metrics and the individual metrics (MI and SR). We adopt a specific form of precoder, such that both MI and SR can be transformed into convex metrics.

Notations: a denotes a vector, A denotes a matrix, and italic English letters like *N* and lower-case Greek letters α are scalar. $|A|,A^{T},A^{H},A^{*},$ and $A^{†}$ represent determinant value, transpose, conjugate transpose, conjugate, and pseudo inverse, respectively. We use diag(a) to denote a diagonal matrix with diagonal entries being the entries of a and Tr(A) to denote the trace of a square matrix. ${∥A∥}_{F}$ represents the Frobenius norm of a matrix.

## 2. System Models and Performance Metrics

**Figure 1:**
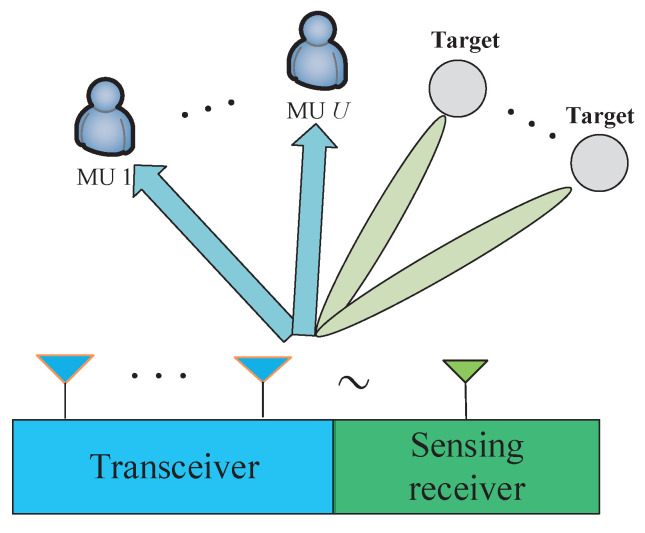
Illustration of the ISAC architecture.

We construct the ISAC architecture, as shown in Figure 1, where a base station communicates with multiple users and meanwhile detects targets. Multiple data streams are sent in parallel from the baseband precoder after digital precoding. The processed signal can realize joint communication and radar functions. At the user receiving end, each user uses a single antenna to receive the information sent by the BS. The BS is equipped with an $N_{T}\times 1$ ULA and an $N_{R}\times 1$ sensing receiver, where $N_{R}\leq N_{T}$ and the multi-antenna sensing receiver is distant from the transmitter to avoid short-range leakage. The data-stream vector at the baseband is denoted as s that is an $N_{S}\times 1$ digital-domain vector and is processed by a baseband digital precoder, P, of dimension $N_{T}\times N_{S}$. We constrain the power of P, such that ${∥P∥}_{F}^{2}\leq P_{o}$, with $P_{o}$ the transmit power. After the digital precoding, the signal is transmitted to the antenna front end and the transmitted signal vector is expressed as

(1) $$\begin{array}{c} x=Ps. \end{array}$$

The transmitted signal impinges on *K* targets and *U* users. At the sensing receiver, the signal received by BS is written as

(2) $$\begin{array}{c} r=G^{H}Ps+n, \end{array}$$

where G is the radar channel matrix of dimension $N_{T}\times N_{R}$ and n is an additive white Gaussian noise (AWGN) vector with zero mean and covariance matrix of $\sigma_{r}^{2}I_{N_{R}}$. When estimating G, multiple data streams are formed into a data block. Here, we let the data block equal $S=[s_{1},\cdots ,s_{L}]$, with each $s_{l}$ the data stream vector of the *l*th time slot. Then, the received signal block is given by

(3) $$\begin{array}{c} R=G^{H}PS+N, \end{array}$$

where R has a dimension of $N_{R}\times L$ and N is the corresponding noise matrix. We adopt a geometric channel model for radar, that is, each target denotes one non-line-of-sight path. The channel model is expressed as $G^{H}=\sum_{k=1}^{K}g_{k}a_{R}\left(\theta_{k}\right)a_{T}^{H}\left(\theta_{k}\right)$, where $g_{k}$ is the path gain of the *k*th target, $a_{R}\left(\theta_{k}\right)$ and $a_{T}\left(\theta_{k}\right)$ are the array steering vectors at the receiver and transmitter, respectively, and $\theta_{k}$ is the angle between BS and the target.

The MI between the radar channel and the transpose of the received signal, $R^{T}$, can be derived as:

(4) $$\begin{array}{c} \mathrm{MI}=I\left(G;R^{T}|S\right)=\log\left|I_{L}+\frac{1}{\sigma_{r}^{2}N_{R}}S^{T}P^{T}\Sigma_{G}P^{*}S^{*}\right|. \end{array}$$

See proofs in Appendix A.

At each user’s side, the *u*th user’s received signal is written as

(5) $$\begin{array}{c} y_{u}=h_{u}^{H}Ps+n_{u}, \end{array}$$

where $h_{u}$ is the communication channel matrix between BS and the *u*th user, and $n_{u}$ is an AWGN with zero mean and covariance of $\sigma_{c}^{2}$. We adopt a Rayleigh Gaussian channel model for communications, that is, the channel entries between BS and each UE yield a Gaussian distribution of zero mean and variance of 1.

The SINR of each user is given by

(6) $$\begin{array}{cc} {\mathrm{SINR}}_{u}= & \frac{\frac{P_{o}}{U}{\left|{\left(h_{u}\right)}^{H}p_{u}\right|}^{2}}{\frac{P_{o}}{U}\sum_{v\neq u}{\left|{\left(h_{u}\right)}^{H}p_{v}\right|}^{2}+\sigma_{c}^{2}}=\frac{\frac{P_{o}}{U}\mathrm{Tr}\left(Q_{u}H_{u}\right)}{\frac{P_{o}}{U}\sum_{v\neq u}\mathrm{Tr}\left(Q_{v}H_{u}\right)+\sigma_{c}^{2}}, \end{array}$$

where $p_{u}$ ($p_{v}$) is the *u*th (*v*th) column of P, $H_{u}=h_{u}{\left(h_{u}\right)}^{H},Q_{u}=p_{u}p_{u}^{H}$, and $Q_{v}=p_{v}p_{v}^{H}$. The SR of each user is expressed as:

(7) $$\begin{array}{c} {\mathrm{SR}}_{u}=\log_{2}\left(1+{\mathrm{SINR}}_{u}\right). \end{array}$$

It is noted that both MI and SR can be individually optimized with the maximum values given by ${\mathrm{MI}}^{★}$ and ${\mathrm{SR}}^{★}$, respectively. Each user’s optimal SR is denoted as ${\mathrm{SR}}_{u}^{★}$. Clearly, the ISAC waveform cannot achieve both optimal values at the same time. The trade-off point for P should be close to the optimal coordinate, $\left({\mathrm{MI}}^{★},{\mathrm{SR}}^{★}\right)$.

This paper aims to compare one-threshold constrained methods and two-threshold constrained methods. The one-threshold methods optimize one performance metric (either radar MI or communication SR) and constrain the other metric using a threshold. The two-threshold methods constrain two performance metrics and gradually increase the thresholds to narrow the feasible set. Compared with one-threshold (either communication-constrained or radar-constrained) methods, the two-threshold constrained methods have a smaller feasible set but the feasible set approaches to the optimal coordinate, $\left({\mathrm{MI}}^{★},{\mathrm{SR}}^{★}\right)$.

## 3. Proposed Optimization Schemes

In this section, we propose three optimization methods. The first two methods are one-threshold constrained methods and the third method is a two-threshold constrained method. The first two methods can obtain better performance for the optimization goal but the complexity would be higher too. As for the third method, with introducing two thresholds, the obtained solution becomes sub-optimal but we adopt a specific form of the precoder, such that both MI and SR can be simplified and convex toolboxes are enabled to solve the third optimization problem.

### 3.1. Case 1: Communication SR-Constrained Optimization

In the first case, we formulate the optimization problem by maximizing the radar MI and constraining the SR of communication. The SR-constrained problem is expressed as

(8) $$\begin{array}{cc} \arg\; & \max_{P}\mathrm{MI}\\ s.t. & {∥P∥}_{F}^{2}\leq P_{o},{\mathrm{SR}}_{u}\geq \beta_{u}, \end{array}$$

where $\beta_{u}$ is the performance threshold for each user.

The constraint, ${\mathrm{SR}}_{u}\geq \beta_{u}$, is non-convex but it can be equivalently transformed into

(9) $$\begin{array}{cc} J_{u}= & \mathrm{Tr}\left(Q_{u}H_{u}^{H}\right)-\gamma_{u}\left(\sum_{v\neq u}\mathrm{Tr}\left(Q_{v}H_{u}\right)+\frac{\sigma_{c}^{2}U}{P_{o}}\right)\\ = & p_{u}^{H}\left(H_{u}-\gamma_{u}\sum_{v\neq u}H_{v}\right)p_{u}-\frac{\sigma_{c}^{2}U}{P_{o}}\geq 0, \end{array}$$

where $\gamma_{u}=2^{\beta_{u}}-1$. The transformed optimization problem is:

(10) $$\begin{array}{cc} \arg\; & \max_{P}\mathrm{MI}\\ s.t. & {∥P∥}_{F}^{2}\leq P_{o},J_{u}\geq 0. \end{array}$$

Even though $J_{u}$ is still a non-convex function, it has a much simpler form than ${\mathrm{SR}}_{u}$ and traditional convex optimization methodology can be used to guarantee that $J_{u}\geq 0$.

Here, we employ the methodology in [13] and propose an iterative algorithm to optimize the problem above. Before iterations, we need to find a P, such that it satisfies all the constraints. Our proposed Algorithm 1 has two stages.

In the first stage, in each iteration, each column of P, denoted as $p_{s},s=1:N_{S}$, moves in the direction of $g_{s}$, i.e.,

(11) $$\begin{array}{c} p_{s}=p_{s}+Cg_{s} \end{array}$$

where *C* is the scaling coefficient, such that the equation of the constraint is met, $g_{s}=\sum_{x=1}^{X}a_{s,x}v_{x}$, where $v_{x}$ is the *x*th eigenvector of $G^{H}G$, $a_{s,x}$ is a real value to be determined, and *X* is the number of non-zero eigenvalues of $G^{H}G$. The value of $a_{s,x}$ can be determined by maximizing MI without the SR constraint.
**Algorithm 1** Communication constrained ISAC precoder optimization.1:**Input:** G and $h_{u}$.2:**Initialization:** i=0, $\beta_{u}$, and $p_{s}^{\left(0\right)}$ that satifies all constraints.3:Obtain direction vector $g_{s}$.4:**Stage 1:**5:Obtain $p_{s}^{\left(i\right)}$ according to (11).6:**Stage 2:**7:**while** MI keeps rising **do**8:   Obtain $p_{s\pm }^{\left(i\right)}$ according to (12).9:   Scale $p_{s\pm }^{\left(i+1\right)}$, such that the equation of the constraint is met.10:   Select $p_{s}^{\left(i+1\right)}$ from $p_{s\pm }^{\left(i+1\right)}$, such that MI keeps rising.11:**end while**12:**Output:** P.

In the second stage, when the iterative point of $p_{s}$ reaches the equation constraint, $p_{s}$ begins to move along the equation of the constraint, e.g., $J_{u}=0$ or ${∥P∥}_{F}^{2}=P_{o}$, which is realized as follows. We generate two precoding vectors moving in the directions of $g_{s}$.

(12) $$\begin{array}{c} p_{s-}^{\left(i\right)}=p_{s}^{\left(i\right)}-\epsilon g_{s},p_{s+}^{\left(i\right)}=p_{s}^{\left(i\right)}+\epsilon g_{s}, \end{array}$$

where $p_{s}^{\left(i\right)}$ is the *s*th column of P in the *i*th iteration, such that the equation of the constraint is met, and ϵ is a small value. As for $p_{u-}^{\left(i\right)}$ and $p_{u+}^{\left(i\right)}$, they are not on the surface of the constraint, which means the equation of the constraint is not met. Then, we project $p_{s-}^{\left(i\right)}$ and $p_{s+}^{\left(j\right)}$ onto the surface of the constraint, which is simply realized by scaling the modulus of $p_{s-}^{\left(i\right)}$ and $p_{s+}^{\left(i\right)}$, such that ${∥P∥}_{F}^{2}=P_{o}$ or $J_{u}=0$. Either the scaled $p_{u-}^{\left(i\right)}$ or $p_{u+}^{\left(i\right)}$ should make the objective function, MI, keep rising. We select the one that makes MI increase as the next iterative point. Update the iteration index i=i+1 and repeat the same procedure in Stage 2. We terminate the iteration when MI stops rising.

### 3.2. Case 2: Radar MI-Constrained Optimization

In the second case, we formulate the optimization problem by maximizing the minimal SR of all users and constraining the MI of radar. The MI-constrained problem is expressed as

(13) $$\begin{array}{cc} \arg\; & \max_{P}\min_{u}{\mathrm{SR}}_{u}\\ s.t. & {∥P∥}_{F}^{2}\leq P_{o},\mathrm{MI}\geq \lambda , \end{array}$$

where λ is the threshold of MI.

It is noted that SR is not a convex function. Hence, compared with the first case, it is more difficult to optimize the SR. To solve this problem, some relaxing methods, such as semi-definite relaxing (SDR) or geometric relaxing methods [13], can be used to transform the SR into a convex function. We propose a novel algorithm for the problem of (13).

We define $\beta =\min_{u}{\mathrm{SR}}_{u}$ as the smallest SR that is an auxiliary value. The optimization problem is recast as:

(14) $$\begin{array}{cc} \arg\; & \max_{P}\beta \\ s.t. & {∥P∥}_{F}^{2}\leq P_{o},\mathrm{MI}\geq \lambda ,{\mathrm{SR}}_{u}\geq \beta . \end{array}$$

It is noted that SINR (SR) is more suitable to be treated as a constraint rather than the objective function in an optimization problem to avoid a non-convex objective function.

Without the MI constraint, the problem above can be solved directly using SDP, which can be referred to [14]. However, when including the MI constraint, we note that:

(15) $$\begin{array}{c} \mathrm{MI}\neq \log_{2}\left|I+\sigma_{r}^{-2}N_{R}^{-1}G^{H}PE\left\{SS^{H}\right\}P^{H}G\right|. \end{array}$$

Only the right-hand side of (15) can be written as the function of $Q=PP^{H}$, which enables the SDP to optimize the problem. However, MI cannot be written as the function of Q, which incurs troubles for SDP to optimize (14). Some alternative algorithms need to be obtained through alternating optimization.

Here, we propose an iterative algorithm to optimize the problem above. Before iterations, we need to find a P that satisfies all the constraints. Note that ${\mathrm{SR}}_{u}\geq \beta$ is equivalent to $J_{u}\geq 0$, where all $\gamma_{u}$ equal γ in $J_{u}$.

Our proposed Algorithm 2 also has two stages. In the first stage, in each iteration, each column of P, $p_{s},s=1:N_{S}$, moves in the direction of $b_{s}$, i.e.,

(16) $$\begin{array}{c} p_{s}=p_{s}+Cb_{s} \end{array}$$

where $b_{s}=\sum_{u=1}^{U}b_{s,u}c_{u}$, where $c_{u}$ is the positive eigenvector of $H_{u}-\gamma_{u}\sum_{v\neq u}H_{v}$, $b_{s,u}$ is a real value to be determined. The value of $b_{s,u}$ can be determined by maximizing β without the MI constraint.
**Algorithm 2** Radar constrained ISAC precoder optimization.1:**Input:** G and $h_{u}$.2:**Initialization:** i=0, λ, and $p_{s}^{\left(0\right)}$ that satisfies all constraints.3:Obtain direction vector $b_{s}$.4:**Stage 1:**5:Obtain $p_{s}^{\left(i\right)}$ according to (16).6:**Stage 2:**7:**while** β keeps rising **do**8:   Obtain $p_{s\pm }^{\left(i\right)}$ according to (17).9:   Scale $p_{s\pm }^{\left(i+1\right)}$, such that the equation of the constraint is met.10:   Select $p_{s}^{\left(i+1\right)}$ from $p_{s\pm }^{\left(i+1\right)}$, such that β keeps rising.11:**end while**12:**Output:** P.

In the second stage, when the iterative point of $p_{s}$ reaches one of the equation constraints, $p_{s}$ begins to move along the equation of the constraint, e.g., $J_{u}=0$ or ${∥P∥}_{F}^{2}=P_{o}$ or MI=λ, which is realized as follows. We generate two precoding vectors moving in the directions of $b_{s}$.

(17) $$\begin{array}{c} p_{s-}^{\left(i\right)}=p_{s}^{\left(i\right)}-\epsilon b_{s},p_{s+}^{\left(i\right)}=p_{s}^{\left(i\right)}+\epsilon b_{s}, \end{array}$$

where $p_{s}^{\left(i\right)}$ is the *s*th column of P in the *i*th iteration, such that the equation of the constraint is met, and ϵ is a small value. As for $p_{u-}^{\left(i\right)}$ and $p_{u+}^{\left(i\right)}$, they are not on the surface of the constraint, which means the equation of the constraint is not met. Then, we project $p_{s-}^{\left(i\right)}$ and $p_{s+}^{\left(j\right)}$ onto the surface of the constraint, which is simply realized by scaling the modulus of $p_{s-}^{\left(i\right)}$ and $p_{s+}^{\left(i\right)}$, such that ${∥P∥}_{F}^{2}=P_{o}$ or $J_{u}=0$. Either the scaled $p_{u-}^{\left(i\right)}$ or $p_{u+}^{\left(i\right)}$ should make the objective function, β, keep rising. We select the one that makes MI increase as the next iterative point. Update the iteration index i=i+1 and repeat the same procedure in Stage 2. We terminate the iteration when β stops rising.

### 3.3. Case 3: ISAC Constrained Optimization

In the third case, we formulate the optimization problem by constraining both the MI of radar and the SR of communication. The optimization goal would be the Euclidean distance between the thresholds and the optimal coordinate. The ISAC-constrained problem is expressed as:

(18) $$\begin{array}{cc} \arg\; & \min_{P}{\left(\lambda -{\mathrm{MI}}^{★}\right)}^{2}+{\left(\beta -{\mathrm{SR}}^{★}\right)}^{2}\\ s.t. & {∥P∥}_{F}^{2}\leq P_{o},\mathrm{MI}\geq \lambda ,J_{u}\geq 0. \end{array}$$

We propose a new algorithm to solve the problem. We define

(19) $$\begin{array}{c} \left[q_{1},\cdots ,q_{n},\cdots ,q_{X+U}\right]={\left[h_{1},\cdots ,h_{U},v_{1},\cdots ,v_{X}\right]}^{†}, \end{array}$$

where $v_{x}$, x=1:X, is the eigenvector of $GG^{H}$ with non-zero eigenvalues. If $X+U<N_{T}$, the vectors, $q_{U+X+1},\cdots ,q_{N_{T}}$, form the null-space of $\left[q_{1},\cdots ,q_{X+U}\right]$. Then, we let all $q_{n},n=1:N_{T}$, be normalized, i.e., $∥q_{n}∥=1$.

We let

(20) $$\begin{array}{c} p_{s}=\sum_{n=1}^{N_{T}}d_{s,n}q_{n},s\in \left\{1,\cdots ,N_{S}\right\}, \end{array}$$

where $d_{s,n}$ is an auxiliary value. Note that $q_{n}$ are $N_{T}$ linearly independent vectors, thus, $p_{s}$ is an arbitrary vector in the whole space $C^{N_{T}\times 1}$. We substitute (20) into the constraints of (18) and suppose that the precoding vectors are nearly orthogonal with each other. The first constraint becomes:

(21) $$\begin{array}{c} \sum_{s=1}^{N_{S}}\sum_{n=1}^{N_{T}}{\left|d_{s,n}\right|}^{2}\leq P_{o}. \end{array}$$

The second constraint approximately becomes:

(22) $$\begin{array}{c} \sum_{n=1+U}^{U+X}\sigma_{r}^{-2}P_{o}{\left|d_{s,n}\right|}^{2}G_{n}^{2}+1\geq 2^{\bar{\lambda }}, \end{array}$$

where $G_{n}$ is the eigenvalue of $GG^{H}$ and $\bar{\lambda }=\lambda /X$ is the average MI for each stream. The third constraint approximately becomes:

(23) $$\begin{array}{c} |d_{u,u}{|}^{2}{∥h_{u}∥}_{F}^{2}-\gamma \left(\sum_{v\neq u}|d_{v,u}{|}^{2}{∥h_{u}∥}_{F}^{2}+\frac{\sigma_{c}^{2}U}{P_{o}}\right)\geq 0, \end{array}$$

where $\gamma =2^{\beta }-1$. Letting $A_{s,n}={∥d_{s,n}∥}^{2}$ and $\mu =2^{\bar{\lambda }}$, we transform the problem of (18) into a convex problem:

(24) $$\begin{array}{cc} \arg\; & \min_{d_{s,n}}{\left(\mu -2^{{\mathrm{MI}}^{★}}{)}^{2}+(\gamma -2^{{\mathrm{SR}}^{★}}+1\right)}^{2}\\ s.t. & \left(21\right),\left(22\right),\left(23\right),A_{s,n}\geq 0. \end{array}$$

The convex problem is only related to the scalars, including $A_{s,n}$, μ, and γ. Hence, it can be solved using traditional convex toolboxes.

## 4. Simulation Results

In this section, we provide simulation results to validate the proposed optimization methods, using numerical experiments on MATLAB. The system parameters are detailed in Table 1 unless mentioned specifically. The BS adopts a 16×1 ULA as the transmit antenna and transmits a 4×1 vector, s. Each user only receives one out of four streams. The remaining two streams carry no information. The angles between BS and each target are randomly distributed in (−π,π).

**Figure 2:**
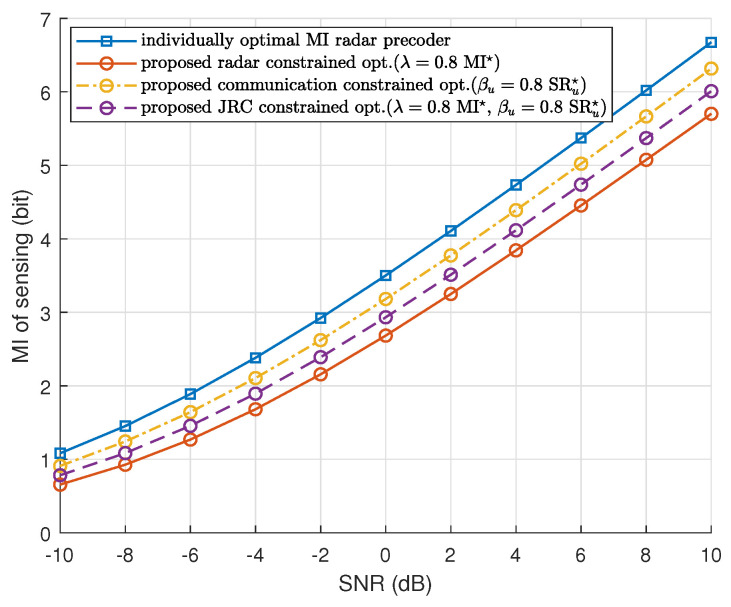
MI of radar versus SNR using the proposed three optimization methods, compared with the individually optimal radar MI precoder.

Figure 2 shows the MI of radar versus SNR for the proposed waveform optimization methods. The SNR is defined as $P_{o}/\sigma^{2}$ with $\sigma =\sigma_{r}=\sigma_{c}$. The benchmark is the individually optimal radar precoder that is obtained by maximizing the MI of radar without communication constraints. The MI of the benchmark remains the highest, which is as expected. We see that the MI-constrained method remains lower than the other two proposed methods, i.e., SR (communication) constrained and ISAC-constrained methods. This is because we selected a relatively low threshold, that is, 0.8 times the optimal solutions for both λ and $\gamma_{u}$. In this case, the MI-constrained method will keep achieving the MI at a relatively low level and biasedly optimize the communication SR. As for the ISAC-constrained method, it performs worse than the SR-constrained method, which is as expected since it has one extra constraint than the SR-constrained optimization.

**Figure 3:**
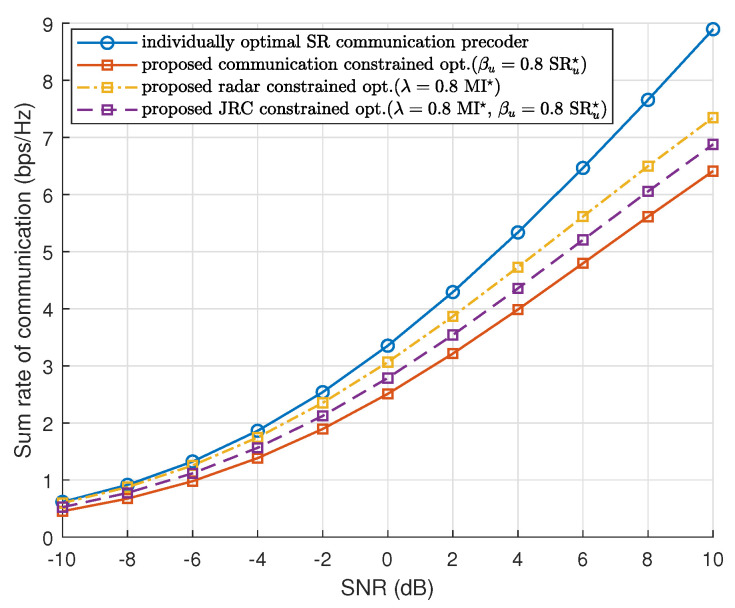
Sum rate of communication versus SNR using the proposed three optimization methods, compared with the individually optimal communication SR precoder.

Figure 3 plots the SR of communication versus SNR for the proposed waveform optimization methods. From the figure, we can obtain nearly the same conclusions as Figure 2. For the individually optimal communication precoder, the SR remains the highest because it is optimized for communication only. We see that the SR-constrained method achieves lower SR than the other two optimization methods because we selected 0.8 times the optimal solutions for both λ and $\gamma_{u}$, which means that the SR-constrained optimization keeps achieving the SR at the level of 0.8 times the optimal value and biasedly optimizes the radar MI. Compared with Figure 2, the SR-constrained method obtains higher MI and the MI-contained method obtains higher SR. As for the ISAC-constrained method, it makes a trade-off between the MI- and the SR- constrained method. This indicates that the ISAC-constrained method can guarantee performance when setting an inappropriate threshold.

**Figure 4:**
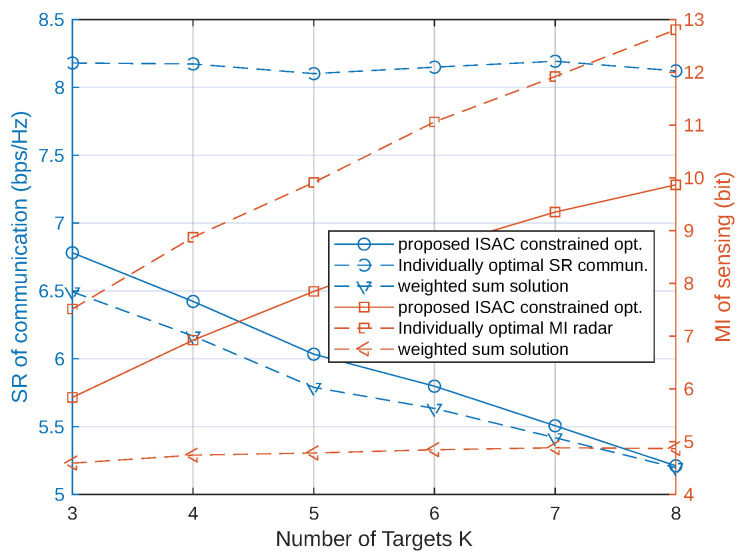
MI and SR versus the number of targets *K* using the proposed ISAC optimization methods, compared with the individually optimal precoder and weighted-sum solution in [8].

Figure 4 unfolds the impacts of *K* on the MI of radar and SR of communications. We compare our proposed ISAC optimization method with the individually optimal precoders and weighted-sum solution in [8]. For our scheme, we let $N_{S}=\max\left(K,U\right)$ in order to improve the system performance. The power of the precoders of all methods are normalized to $P_{o}$. The number of UEs is fixed as 2. For communication, we see that the individually optimal SR remains unchanged since the precoder is not influenced by radar targets. Both our method and the weighted-sum solution achieves lower SR with *K* increasing, which is because the radar channel becomes dominant in the ISAC channels. As for radar, we see that the individually optimal MI keeps rising with *K*. The MI of our method increases with *K* too and can approach to the individually optimal MI. The weighted-sum solution requires that the optimal radar and communication precoders have the same size. Due to the mismatch between *K* and *U*, we see that the MI of the weighted-sum solution is far lower than our achieved MI. It should be noted that the MI and SR are nearly symmetric metrics, and thus, the system performance versus *U* can be deducted by using the conclusion of this figure.

## 5. Conclusions

We have proposed three optimization methods based on the threshold-constrained methodology. The one-threshold (SR/MI-constrained) methods tackle the non-convex optimization problem due to the non-convex nature of MI and SR, whereas the doubly-threshold (ISAC-constrained) method uses traditional convex toolboxes to optimize the waveform. In the one-threshold constrained method, the searching area is larger than that of the doubly-threshold constrained method. Simulation results show that the SR-constrained method obtains higher MI and the MI-contained method obtains higher SR. The doubly constrained method achieves a balanced performance between the MI of radar and the SR of communications.

## Figures and Tables

**Table 1:** Setups of system parameters.

Parameter	Value	Parameter	Value
$N_{T}$	16	$N_{R}$	8
*K*	3	*U*	2
$N_{S}$	4	$P_{o}$	2

## Data Availability

Not applicable.

## Abbreviations

The following abbreviations are used in this manuscript:

AWGN	Additive white Gaussian noise
BS	Base station
ISAC	Integrated sensing and communication
MI	Mutual information
SINR	Signal-to-interference-plus-noise ratio
SDR	Semi-definite programming
SR	Sum rate
SNR	Signal-to-noise ratio

## Appendix A. Derivations for MI of Radar

We note that $R^{T}∼\mathrm{CN}\left(0,D\right)$ with $D=S^{T}P^{T}\Sigma_{G}P^{*}S^{*}+\sigma^{2}N_{R}I_{L}$ and $\Sigma_{G}=E\left\{G^{*}G^{T}\right\}$. The conditional MI between G and received sensing signal block, R, is calculated as

(A1) $$\begin{array}{c} I\left(G;R^{T}|S\right)=h\left(R^{T}|S\right)-h\left(N^{T}\right), \end{array}$$

where h(·) is the entropy. Then, we need to obtain the conditional probability density function (pdf) of $R^{T}$ for a given S, given by:

(A2) $$\begin{array}{c} \mathrm{pdf}\left(R^{T}|S\right)=\frac{1}{\pi^{N_{R}L}|\sigma_{r}^{2}N_{R}I_{L}+S^{T}P^{T}\Sigma_{G}P^{*}S^{*}|}\exp\left(-\mathrm{Tr}\left(R^{T}D^{-1}R^{*}\right)\right). \end{array}$$

Then, $h(R^{T}|S)$ is calculated as:

(A3) $$\begin{array}{cc} h(R^{T}|S)= & N_{R}L\log\left(\pi \right)+\log\left|\sigma_{r}^{2}I_{L}N_{R}+S^{T}P^{T}\Sigma_{G}P^{*}S^{*}\right|+E\left(\mathrm{Tr}\left(R^{T}D^{-1}R^{*}\right)\right)\\ = & N_{R}L\log\left(\pi \right)+\log\left|\sigma_{r}^{2}I_{L}N_{R}+S^{H}P^{H}\Sigma_{G}PS\right|+L. \end{array}$$

The entropy of noise is given by:

(A4) $$\begin{array}{cc} h(N)= & N_{R}L\log\left(\pi \right)+\log\left|\sigma_{r}^{2}I_{L}N_{R}\right|+L. \end{array}$$

Then, the MI is given by:

(A5) $$\begin{array}{c} I\left(G;R^{T}|S\right)=\log\left|I_{L}+\frac{1}{\sigma^{2}N_{R}}S^{T}P^{T}\Sigma_{G}P^{*}S^{*}\right|. \end{array}$$
